# Integrated Counts of Carbohydrate-Active Protein Domains as Metabolic Readouts to Distinguish Probiotic Biology and Human Fecal Metagenomes

**DOI:** 10.1038/s41598-019-53173-7

**Published:** 2019-11-14

**Authors:** Hong-Hsing Liu, Yu-Chen Lin, Chen-Shuan Chung, Kevin Liu, Ya-Hui Chang, Chung-Hsiang Yang, Yun Chen, Yen-Hsuan Ni, Pi-Feng Chang

**Affiliations:** 1 Institute of Molecular and Genomic Medicine, National Health Research Institutes, Zhunan Town, Miaoli County 350 Taiwan; 2 Pediatrics, En Chu Kong Hospital, Sanxia District, New Taipei City, 237 Taiwan; 3 Pediatrics, Far Eastern Memorial Hospital, Pan-Chiao District, New Taipei City, 220 Taiwan; 4 Electronic Engineering, Oriental Institute of Technology, Pan-Chiao District, New Taipei City, 220 Taiwan; 5 Internal Medicine, Far Eastern Memorial Hospital, Pan-Chiao District, New Taipei City, 220 Taiwan; 6 Pediatric Surgery, Far Eastern Memorial Hospital, Pan-Chiao District, New Taipei City, 220 Taiwan; 7 Pediatrics, National Taiwan University Hospital, Zhongzheng District, Taipei, 100 Taiwan

**Keywords:** Microbiota, Sequence annotation

## Abstract

Bowel microbiota is a “metaorgan” of metabolisms on which quantitative readouts must be performed before interventions can be introduced and evaluated. The study of the effects of probiotic *Clostridium butyricum* MIYAIRI 588 (*CBM588*) on intestine transplantees indicated an increased percentage of the “other glycan degradation” pathway in 16S-rRNA-inferred metagenomes. To verify the prediction, a scoring system of carbohydrate metabolisms derived from shotgun metagenomes was developed using hidden Markov models. A significant correlation (R = 0.9, *p* < 0.015) between both modalities was demonstrated. An independent validation revealed a strong complementarity (R = −0.97, *p* < 0.002) between the scores and the abundance of “glycogen degradation” in bacteria communities. On applying the system to bacteria genomes, *CBM588* had only 1 match and ranked higher than the other 8 bacteria evaluated. The gram-stain properties were significantly correlated to the scores (*p* < 5 × 10^−4^). The distributions of the scored protein domains indicated that *CBM588* had a considerably higher (*p* < 10^−5^) proportion of carbohydrate-binding modules than other bacteria, which suggested the superior ability of *CBM588* to access carbohydrates as a metabolic driver to the bowel microbiome. These results demonstrated the use of integrated counts of protein domains as a feasible readout for metabolic potential within bacteria genomes and human metagenomes.

## Introduction

Bowel microbiota are now considered “metaorgans”^1^ for humans in which bacteria occupy a considerable proportion^2^. Various functions are associated with these microbes. For example, gut immune maturation depends on colonization with a host-specific microbiota^3^, but an abundance of *Ruminococcus gnavus* is related to allergic diseases in infants^4^. In addition to immune interactions with hosts, these microbes contribute substantially to metabolic processes in the bowel. For example, short chain fatty acids are crucial energy sources produced by bacteria^5^ but are also pathologically related to the metabolic syndrome in humans^6^. Due to the multifaceted roles of this metaorgan, informative readouts are crucial for evaluating its metabolic potential.

Recent advances in sequencing technology^7^ have enabled the in-depth taxonomic profiling of gut microbiota. Signatures from 16S subunits of ribosomes have made the culture-free categorization of bacteria possible^8^. However no practical markers are available to quantify metabolic functions. Tools such as PICRUSt^9^ or Piphillin^10^ can be extrapolated to identify metabolic profiles by mapping the characteristic 16S sequences to known reference genomes. Alternatively, bioinformatic pipelines can directly interpret shotgun metagenomes^11^. However most of these pipelines require specialized programs or a series of tools to yield results that are difficult to interpret for individuals without specialized knowledge such as patients. A system that summarizes the metabolic profile of this metaorgan in formats conveyable to both nonexperts and experts is highly desirable for streamlining the use of interventions such as courses of prebiotics or probiotics and fecal material transfer.

We conducted a pilot study examining *Clostridium butyricum* Miyairi 588 (*CBM588*) in patients undergoing small bowel transplantation (SBT). Although the survival rate of patients after SBT is now 70%^12^ with the assistance of optimized immunosuppressants, further improvements could be made. Studies have found that bacteria diversities in bowels can confer a favorable prognosis factor in patients undergoing allogeneic hematopoietic stem cell transplantation^13^. Patients who do and do not reject SBTs do have different compositions of ileal microbiota^14^. A proactive measure to control gut microbiota could be a valuable addition to the care of SBT patients. Probiotics could be a promising option in suitable candidates. Based on these considerations and the results of 16S-based taxonomic and functional analyses, we successfully developed a scoring system that not only had favorable correlations with 16S-based reports but also offered mechanistic insights into how *CBM588* drives the evolution of fecal bacteria communities in SBT recipients. The scores were based on integrated counts of carbohydrate-active protein domains after probability analyses were conducted using hidden Markov models. This system indicates the potential of protein domain–based scoring of focused metabolisms as readouts for understanding probiotic characteristics and their effects on fecal metagenomes.

## Results

### Taxonomic shifts of fecal microbiota associated with *CBM588* ingestion

**Figure 1:**
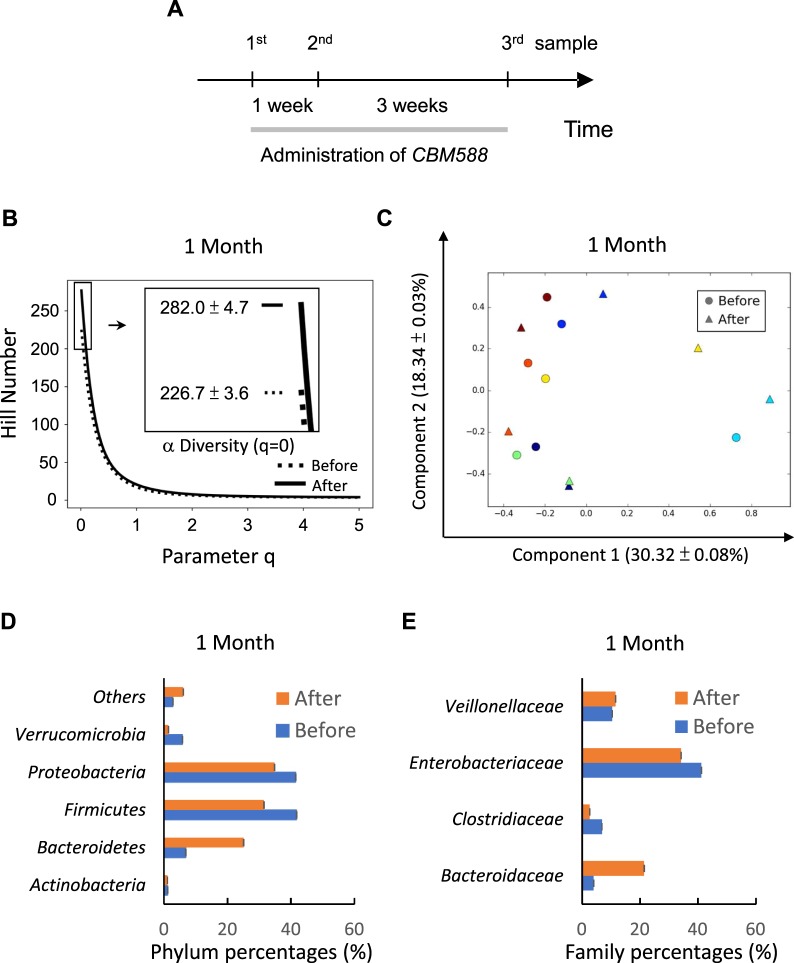
Study design and taxonomic evaluations of fecal microbiota associated with *CBM588* ingestion for 1 month. (**A)** Patients took *CBM588* continuously for 1 month. Stool samples were collected before, 1 week after, and 1 month after the administration of *CBM588*. **(B)** OTU diversities at 1 month were assayed using *q*-parameterized Hill numbers. No OTUs were dominant after *CBM588* administration, as suggested by overlapping dashed (before administration) and solid (1 month after administration) curves. α diversity (q = 0) was increased from 226.7 ± 3.6 to 282.0 ± 4.7 (SD), as averaged from 10 rarefied data sets. **(C)** The administration of *CBM588* for 1 month caused discernible changes in the OTU profiles of all patients according to principal component analyses. Circles (before administration) and triangles (1 month after administration) of the same color were separated. **(D)** Percentages of 5 phyla, namely *Actinobacteria, Bacteroidetes, Firmicutes, Proteobacteria, and Verrucomicrobia*, shifted significantly (*p* < 0.05) according to mixed linear models of at least half of the rarefied data sets after the administration of *CBM588* for 1 month. **(E)** 4 families were selected after patients received *CBM588* for 1 month by intersecting the results of both support vector classification and logistic regression models among rarefied data sets. *Bacteroidaceae*, *Enterobacteriaceae*, and *Veillonellaceae* were evident among all rarefactions, whereas *Clostridiaceae* was noted in half of the data sets.

We recruited 7 patients 6 months after their small bowel transplantations (SBTs) (Table 1; 3 males and 4 females). They took oral *CBM588* (1.5 × 10^9^ CFU/day) daily for 1 month (Fig. 1A). The median age was 37 years (range, 16–59 years). Stool samples were collected before, 1 week after, and 1 month after *CBM588* ingestion. Microbiota were profiled by sequencing 16S rRNA-based amplicons^8^ with a paired-end approach (raw read numbers are presented in Supplementary Fig. S1A). Operational taxonomic units (OTUs) were defined using a USEARCH-based pipeline^15^. Rarefaction curves of distinct OTUs were constructed by administering 10 random samplings for each patient (Supplementary Fig. S1B). At each depth, the averages of unique OTUs were plotted. All rarefaction curves showed saturating behaviors with an increase in read depths. To compensate for uneven read numbers among different samples, 10 randomly rarefied data sets with normalized reads in mapped OTU format were prepared from the original data sets before downstream analyses were conducted (Supplementary Fig. S2A). These 7 SBT recipients experienced no apparent infection or rejection during the study period.

**Table 1:** Clinical characteristics of 7 SBT patients.

ID	Sex	Age	Diagnosis
P1	Female	20	Intestinal failure due to megacystis microcolon intestinal hypoperistalsis syndrome
P2	Male	59	Short bowel syndrome after occlusion of the superior mesenteric artery
P3	Female	16	Short bowel syndrome after massive resection of paraduodenal hernia
P4	Female	55	Short bowel syndrome after massive resection for adhesion lysis
P5	Male	57	Short bowel syndrome after massive bowel resection for gastrointestinal stromal tumor
P6	Male	37	Intestinal failure due to chronic intestinal pseudo-obstruction syndrome
P7	Female	34	Short bowel syndrome after massive resection for adhesion lysis

Sample diversities were assayed in units of OTU. Those at the same time point were pooled and averaged before analyses. Hill numbers^16^ were adopted to evaluate diversities with a parameter *q* (Fig. 1B and Supplementary Fig. S3A). With increasing values of *q*, the contributions of OTU abundances were increasingly weighted in results of Hill number-based diversities. Without weights (*q* = 0), Hill numbers were equal to α diversities (see Methods). All 10 rarefied data sets were evaluated and exhibited almost identical plots (Supplementary Fig. S4). After 1 week, α diversities positively built up from 226.3 ± 2.6 to 251.0 ± 3.2 (SD), representing a 10.9% increase (Supplementary Fig. S3A). The trend continued further at 1 month (Fig. 1B), increasing by 24.3% from 226.7 ± 3.6 to 282.0 ± 4.7 (SD). However, no significant differences in profiles were noted from positive-*q* Hill numbers after *CBM588* ingestions for either 1 week or 1 month, implying the absence of dominant OTUs in samples.

Contrasts between samples were summarized using principal component analyses (PCAs). For each sample, percentages of OTUs were Hellinger-transformed before analyses were conducted^17^. All 10 rarefied data sets were evaluated, and few differences were noted among the plots (Supplementary Fig. S5). Supplementary Fig. S3B and Fig. 1C present representative 1-week and 1-month results, respectively. With variances of 28.80 ± 0.03% and 14.85 ± 0.03% (SD) explained by PCA leading components, we revealed that 1 week of exposure did not alter microbiota considerably from the baselines (Supplementary Fig. S3B, triangles *vs*. circles of the same color). At 1 month, 30.32 ± 0.08% and 18.34 ± 0.03% (SD) of total variances could be accounted for by the top 2 PCA components. *CBM588* samples were separated from *CBM588*-naïve samples for all patients (Fig. 1C, triangles *vs*. circles of the same color). This time-dependent divergence suggested a *CBM588*-driven effect on the taxonomic profiles of gut microbiota. The inability of PCAs to identify clustering for any of the 3 time points implied considerable individual variations in microbe compositions.

Each OTU was taxonomically classified using USEARCH^15^ with a SILVA-based reference^18^. 16S rRNA reads of each sample were mapped indirectly to a SILVA taxonomy *via* OTU. At the phylum level (Supplementary Fig. S3C and Fig. 1D), 5 (*Actinobacteria*, *Bacteroidetes*, *Firmicutes*, *Proteobacteria*, and *Verrucomicrobia)* out of 10 phyla were demonstrated to have changed significantly (*p* < 0.05) according to mixed linear models^19^ (MLMs) of at least half of the rarefied data sets. Out of the 3 most abundant of these 5 phyla, *Bacteroidetes* increased the most (from 6.84 ± 0.03% to 25.09 ± 0.05% [SD]) after 1 month of *CBM588* administration. *Firmicutes* and *Proteobacteria* decreased from 41.78 ± 0.06% to 31.47 ± 0.05% and 41.53 ± 0.04% to 34.83 ± 0.04% (SD), respectively. Although MLM did not reveal significant changes among any phyla at 1 week, some of the varying trends at 1 month were already discernible at that time point (Supplementary Fig. S3C).

To identify feature families associated with *CBM588* administration, we used support vector classification^20^ (SVC) and logistic regression^21^ (LR) to determine the signature families from contrasts between naïve and 1-month data sets. There were 69 to 74 SILVA-mapped families among the 10 rarefied data sets. Only those that satisfied both SVC and LR models among at least half of the rarefied data sets were selected. We identified *Bacteroidaceae*, *Enterobacteriaceae*, and *Veillonellaceae* among all rarefactions, and *Clostridiaceae* was observed in half of the data sets (Supplementary Fig. S3D and Fig. 1E). At 1 month, the abundance of *Bacteroidaceae* and *Veillonellaceae* increased from 4.00 ± 0.02% to 21.43 ± 0.06% and 10.42 ± 0.03% to 11.55 ± 0.04% (SD), respectively (Fig. 1E). By contrast, the abundance of *Enterobacteriaceae* and *Clostridiaceae* decreased from 41.20 ± 0.04% to 34.16 ± 0.04% and 6.86 ± 0.01% to 2.66 ± 0.02% (SD), respectively. Similar trends were already observable at 1 week (Supplementary Fig. S3D).

### Functional shifts of fecal metagenome associated with *CBM588* ingestion

**Figure 2:**
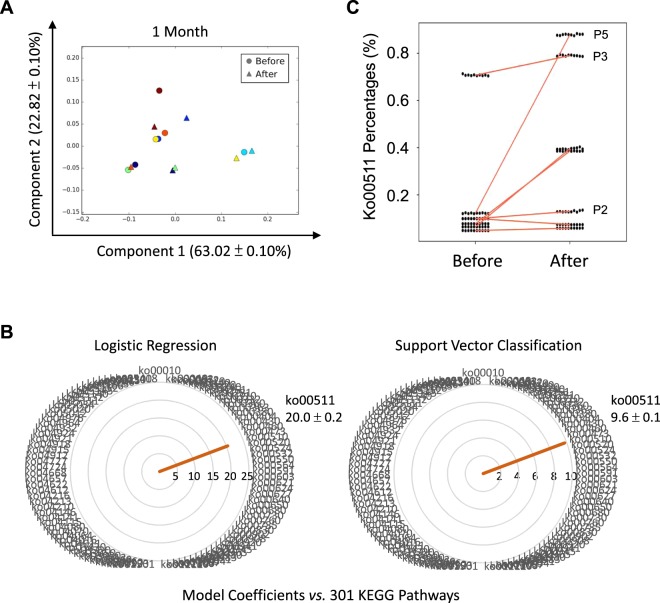
Assessments of fecal metagenome functions with 16S-rRNA. **(A)** PCAs were conducted on profiles of KEGG functional pathways, as inferred by Piphillin with 16S rRNA sequences. Samples before (circles) and 1 month after (triangles) *CBM588* ingestion were plotted. Each patient was designated a specific color. In most patients, apparent shifts after 1 month of exposure were noted. **(B)** Radar plots of 10 rarefactions are prepared with pointing hands as model coefficients from support vector classification and logistic regression, respectively. ko00511 was one and the only pathway agreed by both models. **(C)** Percentages of the ko00511 pathway among 10 rarefied data sets of all samples collected before or 1 month after *CBM588* ingestion are plotted. Red lines connect the same patients. Variations between rarefied data sets were minimal. Nearly all patients had upward trends for the ko00511 pathway.

To investigate metagenome functions from 16S rRNA data sets, all 10 rarefaction data sets were subjected to cross-reference analyses to KEGG pathways^22^ using Piphillin^10^. Variations in associated KEGG pathways were further analyzed using PCA. All rarefied data sets yielded similar plots (Supplementary Fig. S6), with the 1-month administration of *CBM588* causing significant shifts in the plots for most patients (Fig. 2A, triangles *vs*. circles of the same color). The first 2 components explained 63.02 ± 0.10% and 22.82 ± 0.10% (SD) of total variances, respectively. To select feature pathways that were most associated with the 1-month ingestion of *CBM588*, SVC^20^ and LR^21^ models were tested against all rarefied data sets. Only those pathways chosen by both models would be accepted. In each rarefaction, 297 to 301 KEGG pathways were observed; however, only 1 pathway, ko00511 or “other glycan degradation,” was constantly distinct from the others. Figure 1B shows radar plots with model coefficients as pointing hands toward 301 KEGG pathways. The only selected pathway was ko00511 with coefficients of 9.6 ± 0.1 (SD) and 20.0 ± 0.2 (SD) for SVD and LR models, respectively. The corresponding percentages of ko00511 among the samples of all rarefied data sets are displayed in Fig. 2C. Most patients exhibited an upward trend for this “other glycan degradation” pathway with *CBM588* ingestion.

**Figure 3:**
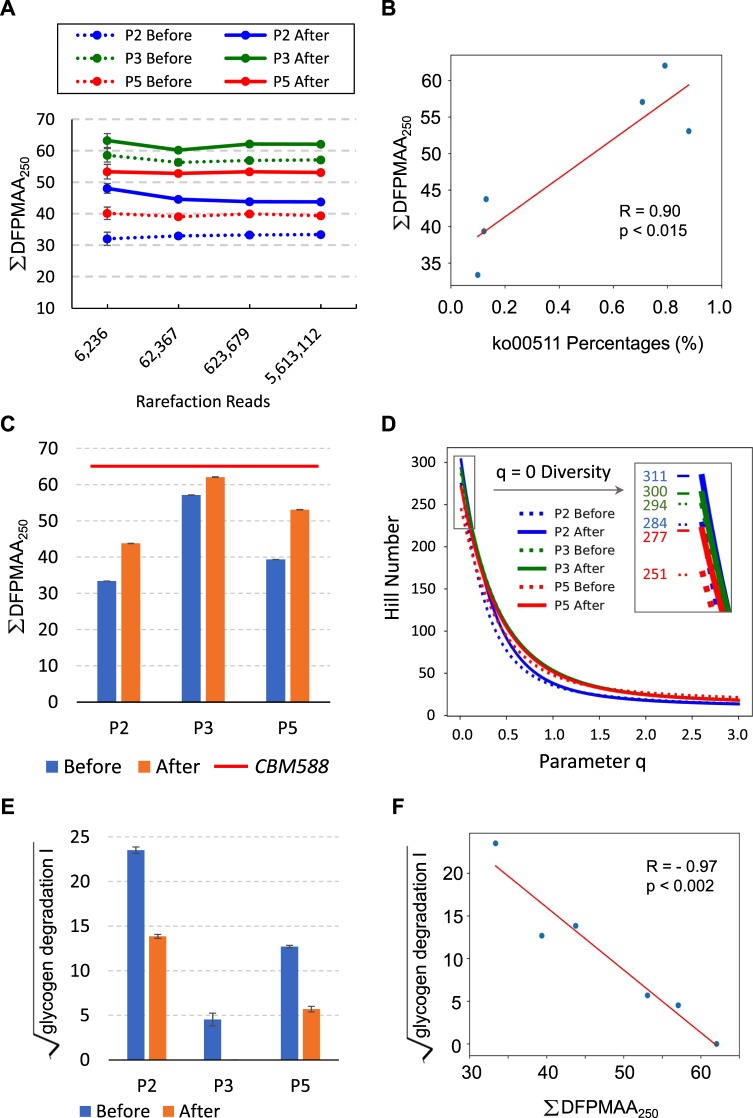
Assessments of fecal metagenome functions with DFPMAA_250_ scores. (**A)** ∑ DFPMAA_250_ values were stable across 3 logs of sequencing depths, as illustrated increasingly from 6,236 to 5,613,112 read numbers. All samples were assayed with 10 rarefied data sets at each depth with error bars in SE. **(B)** ∑DFPMAA_250_ values showed a significant correlation (R = 0.90, *p* < 0.015) with the ko00511 pathway percentages in 16S rRNA-inferred KEGG functional profiles. **(C)** Patients showed an upward trend of ∑DFPMAA_250_ values after 1 month of *CBM588* ingestion. Notably, the *CBM588* genome had higher ∑DFPMAA_250_ scores (red line) than all human fecal metagenomes. **(D)** Diversities of DFPMAA_250_ across all carbohydrate-active protein domains were evaluated with Hill numbers from averages of 10 rarefactions. At q = 0, Hill numbers equal counts of identified domains. The values stepped up from 284 to 311, 294 to 300, and 251 to 277 for P2, P3, and P5, respectively. Mild increases of abundance-weighted diversities were found, especially for P2 and P5 in the parameter range between 0.0 to 1.0 on the q axis. **(E)** Square-rooted abundance (mean ± SE) of “glycogen degradation I” by HUMAnN2 is plotted. Complementarity to corresponding ∑DFPMAA_250_ scores was noted. **(F)** A strong negative correlation (R = −0.97, p < 0.002) between square-rooted quantities of “glycogen degradation I” by HUMAnN2 and summed DFPMAA_250_ scores was evident.

To verify these findings, 6 samples from 3 patients (P2, P3, and P5) with distinct profiles of ko00511 pathway percentages (Fig. 2C) were subjected to the shotgun sequencing of metagenomes. Numbers of raw reads for each sample are listed in Supplementary Fig. S1C. We assumed that the quantities of protein domains involved in carbohydrate processing could serve as indicators of metabolic potential in fecal metagenomes. We employed the dbCAN database^23^ to analyze 585 hidden Markov models (HMMs) of carbohydrate-active domains. Reads from shotgun sequencing were routed to a pipeline to define domain fractions per million amino acids per 250 nucleotides (DFPMAA_250_) for every dbCAN-defined HMM (Supplementary Fig. S2B). The total number of identified domain fractions on a given HMM after normalizations are conducted is DFPMAA_250_ of the given domain. The addition of DFPMAA_250_ across all HMMs was used to estimate overall carbohydrate processing capabilities, (i.e., ∑DFPMAA_250_). Ten rarefied data sets at depths across 3 logs were tested for the robustness of ∑DFPMAA_250_ (Fig. 3A), which revealed a stable trend, especially among depths with read numbers above or equal to 62,367.

We evaluated if estimates of ∑DFPMAA_250_ (Fig. 3A) could exhibit favorable correlations with Piphillin-reported^10^ percentages of the KEGG ko00511 pathway^22^ (Fig. 2C), both of which were averaged from 10 rarefactions. The Pearson coefficient between ∑DFPMAA_250_ and the ko00511 percentages was up to 0.90, which indicated a significant correlation (*p* < 0.015; Fig. 3B). All 3 patients had higher ∑DFPMAA_250_ values after *CBM588* ingestion (*p* < 0.005 after bootstrapping was conducted 10,000 times) (Fig. 3C). The availability of individual DFPMAA_250_ estimates for each HMM enabled the profiling of diversities of carbohydrate-active domains in fecal metagenomes. Hill numbers with varying parameters up to 3.0 were plotted against averages of 10 rarefactions (Fig. 3D), in which higher parameter q values attached more weight to quantitatively dominant domains. Diversities were increased for all 3 patients, especially in the range between 0.0 and 1.0. α diversity, or the zero-*q* Hill number which equals counts of non-zero domains, increased with *CBM588* ingestion from 284 to 311, 294 to 300, and 251 to 277 for P2, P3, and P5, respectively.

To validate above observations, we used HUMAnN2^24^ and MetaCyc^25^ to evaluate the same data sets of shotgun metagenomes (Supplementary Fig. S1C). HUMAnN2 is based on an enhanced search upon known reference genomes, and MetaCyc provides a different catalog of metabolic pathways from KEGG. Analyses were repeated 5 times each with randomly selected 5 million paired reads per sample from original shotgun data sets (Supplementary Fig. S1C). MetaCyc defines “Glycan Degradation” Class with 11 instance pathways. Among the 10 prokaryote-relevant pathways only “glycogen degradation I” was identified by HUMAnN2 in all rarefied data sets. This pathway breaks down intracellular glycogen when carbon sources are limiting^26^. Figure 3E shows the pathway abundance of “glycogen degradation I” in square-rooted units. The values were found to complement ∑DFPMAA_250_ scores (Fig. 3C). The Pearson coefficient between both modalities of assessment was up to −0.97, indicating a strong negative correlation (*p* < 0.002; Fig. 3F).

### Distinct properties of *CBM588* in carbohydrate metabolism

**Figure 4:**
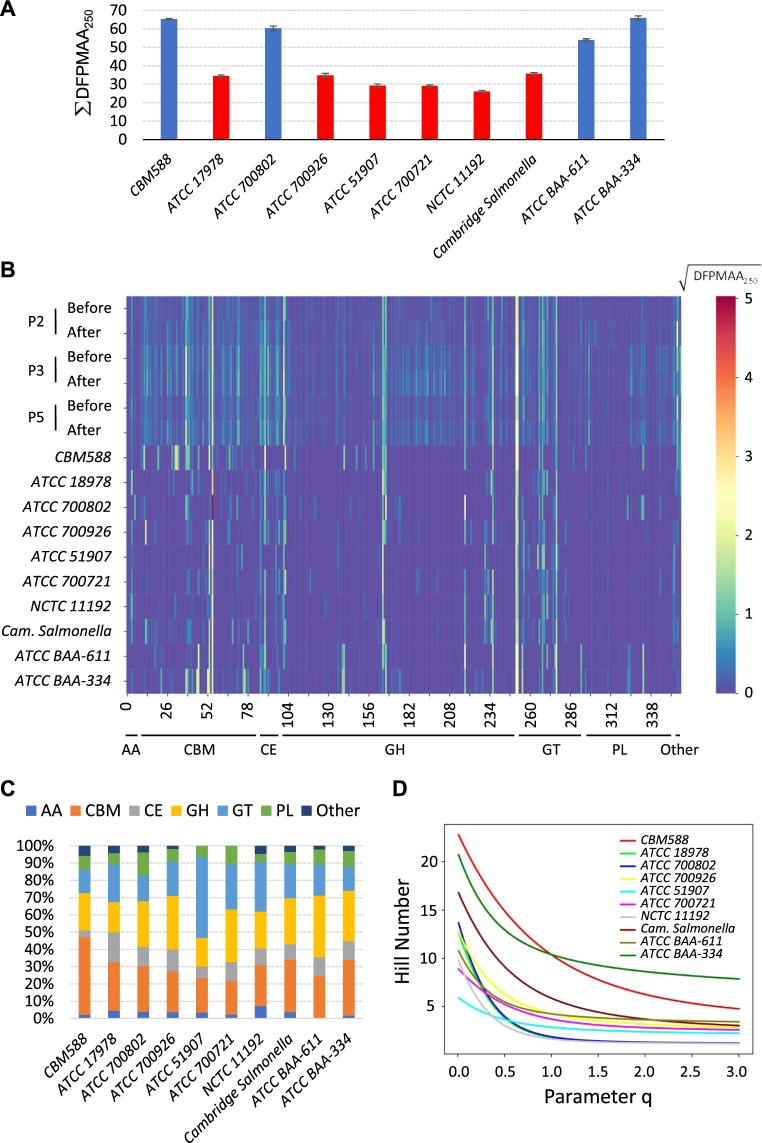
Distinct properties of DFPMAA_250_ scores for *CBM588*. (**A)** The summed DFPMAA_250_ values (∑DFPMAA_250_) across all carbohydrate-active protein domains of the *CBM588* genome was higher than most evaluated bacteria strains. Only 1 strain had a similar value. The gram-positive (blue bars) bacteria had considerably higher estimates than the gram-negative (red bars) bacteria (61.39 ± 2.76 *vs*. 31.64 ± 1.60 [SEM]; *p* < 5 × 10^−4^ according to the Student’s *t*-test). **(B)** The DFPMAA_250_-based heatmap showed a similar distribution pattern for human samples. Different bacteria, however, were equipped with different spectra of carbohydrate-active protein domains encoded in genomes. Domain categories include auxiliary activities (AAs), carbohydrate-binding modules (CBMs), carbohydrate esterases (CEs), glycoside hydrolases (GHs), glycosyltransferases (GTs), polysaccharide lyases (PLs), and others. **(C)** Up to 45% of the *CBM588* carbohydrate-active domains were in the category of CBM, which was considerably higher (*p* < 10^−5^) than the average of the other 9 bacteria (25.42% ± 1.45%, SEM). **(D)** *CBM588* and *ATCC BAA-334* were the two strains with highest CBMs diversities as shown in Hill number profiles. *CBM588* had a steeper curve slope, indicating an unevener distribution of CBMs relative abundances than *ATCC BAA-334*.

We applied the same DFPMAA_250_ pipeline to shotgun sequences of the *CBM588* genome (Supplementary Fig. S2B except Bowtie 2 filters). Notably, ∑DFPMAA_250_ estimated a distinct value for *CBM588* at 66.37 ± 0.13 (SEM), which was higher than any of human samples (Fig. 3C). Because human feces would yield averages from all bowel bacteria, we suspected that most bacteria would carry lower values of ∑DFPMAA_250_ estimates in their genomes. From the data set of Köser *et al*.^27^, we found the shotgun genome sequences of several strains of bacteria (Table 2). To compensate for artificial underestimations of ∑DFPMAA_250_ due to shorter read lengths (Supplementary Fig. S7A), only those bacteria with average paired read lengths longer than 187.6 bp were subjected to DFPMAA_250_ calculation (Table 2 and Supplementary Fig. S7B). Unsurprisingly 8 out of the 9 evaluated bacteria had lower ∑DFPMAA_250_ values than *CBM588*, with 1 being on the same level (*ATCC BAA-334*; Fig. 4A). All Gram-positive bacteria were found to have higher estimates of ∑DFPMAA_250_ values than Gram-negative ones (61.39 ± 2.76 *vs*. 31.64 ± 1.60; SEM; *p* < 5 × 10^−4^ according to *t*-test; Fig. 4A and Table 2).

**Table 2:** Profiles of bacteria strains.

Source	Accessions at ENA*	Species	Gram Stain
*CBM588*	PRJEB27661	*Clostridium butyricum*	Positive
*ATCC 17978*	ERR329997	*Acinetobacter baumannii*	Negative
*ATCC 700802*	ERR330001	*Enterococcus faecalis*	Positive
*ATCC 700926*	ERR330002	*Escherichia coli*	Negative
*ATCC 51907*	ERR330003	*Hemophilus influenza*	Negative
*ATCC 700721*	ERR330004	*Klebsiella pneumoniae*	Negative
*NCTC 11192*	ERR330006	*Legionella pneumophila*	Negative
*Cambridge Salmonella*	ERR330010	*Salmonella enterica*	Negative
*ATCC BAA-611*	ERR330013	*Streptococcus agalactiae*	Positive
*ATCC BAA-334*	ERR330014	*Streptococcus pneumoniae*	Positive

^*^European Nucleotide Archive.

The DFPMAA_250_-based heatmap revealed a similar pattern for all human samples (Fig. 4B), implying a universal repertoire requirement for carbohydrate-active protein domains. Instead the uses and abundances of these protein domains varied considerably among different bacteria (Fig. 4B). We categorized scored counts of families of carbohydrate-active domains^28^ among the 10 strains of bacteria studied, including auxiliary activities (AAs), carbohydrate-binding modules (CBMs), carbohydrate esterases (CEs), glycoside hydrolases (GHs), glycosyltransferases (GTs) and polysaccharide lyases (PLs; Fig. 4C). For *CBM588*, up to 45% of the genome-coded carbohydrate-active domains were in the CBM category, which differed significantly (*p* < 10^−5^) from the other 9 bacteria (25.42% ± 1.45%, SEM). Further characterization of CBMs diversities with Hill numbers (Fig. 4D) revealed that *CBM588* and *ATCC BAA-334* were the two strains with highest values. *CBM588*, however, had a declining curve at a sharper slope, suggesting an unevener distribution of CBMs relative abundances than *ATCC BAA-334*.

## Discussion

DFPMAA_250_ estimates the potentials of carbohydrate metabolisms within bacteria genomes and human metagenomes by integrating counts of relevant protein domains. The underlying assumption is the enzyme amount is the key determinant of chemical reactions within a complex system such as human bowels regardless of the availability of reactants. A system based on a similar logic can successfully predict metabolomic turnover in oceans^29^. With the increasing number of interventions into bowel microbiota for medical benefits, a quantitative base would be required to make logical decisions and predictions for this metaorgan. Our study demonstrates a potential framework for this purpose. Other metabolisms could be formatted similarly. A set of essential scores would provide a valuable reference for clinicians to assess and evaluate bowel microbiomes in the same manner as creatinine for the kidney and transaminases for the liver. Scored summaries would also be easy for non-experts like patients to follow.

Construction of metabolic models between hosts and microbes have been pioneered by many scientists (reviewed by Heinken *et al*.^30^). One category of approaches is “top-down” to find correlations among metabolites by statistical analyses. The other category is “bottom-up” to build metabolic networks with units and coefficients of known pathways plus functional annotations from reference genomes. Designs of “top-down” could help identify novel correlations but often lack mechanistic insights; approaches of “bottom-up” would provide astonishing precision but often take tons of time to calculate. Instead our design can be considered as a middle stop between both ends. Scientists can use domain-based scores as correlation targets to make hypotheses of mechanisms and to design experiments with known properties of the given domains. Involved proteins can be even cloned from nucleotide sequences as mapped to the given HMMs in the data sets. Pathways can be constructed accordingly. These are all advantages not readily available from previous approaches.

Values of ∑DFPMAA_250_ were sensitive to mean read lengths but correctable up to a threshold of approximately 187.6 bp (Supplementary Fig. S7A). This was likely due to an inherent criterion set by dbCAN^23^, which requires covered fractions of HMMs by aligned reads to be higher than 0.3. With 90% of the known protein domains smaller than 200 amino acids^31^, mean read lengths at 187.6 bp would likely be sufficient for calculating DFPMAA_250_ values. Although this would set a limit on available choices of sequencing platforms, the demonstrated insensitivity to sequencing depths (Fig. 3A) could adequately compensate for this disadvantage. With rapid improvements on read lengths from the platform manufacturers, more sequence sets ready for the DFPMAA_250_ pipeline would be expected.

Enhancements of carbohydrate processing capabilities in fecal metagenomes after *CBM588* ingestions were supported by increased ∑DFPMAA_250_ scores (Fig. 3C). These results are compatible with previous findings that *Clostridia* is associated with glycan degradation potential^32^. Because the abundance of the *Clostridiaceae* family decreased in our study (Fig. 1E), *CBM588* had a low likelihood of having a direct contribution by mass effect. Instead, we observed that *CBM588* not only carried a higher overall value of ∑DFPMAA_250_ than other bacteria (Fig. 4A) but also had a significant proportion of carbohydrate-active protein domains in the carbohydrate-binding module category (Fig. 4C). It is likely that *CBM588* indirectly diversifies the microbe community by offering access to more glycan varieties. In addition, the increased *Bacteroidetes* abundance (Fig. 1D) could positively reinforce the glycan-metabolizing potential of the microbiome^33,34^. Independent validation with HUMAnN2^24^ and MetaCyc^25^ identified a negative correlation (Fig. 3F) between pathway abundance of “glycogen degradation I” and ∑DFPMAA_250_ scores. This finding suggests that microbiomes carrying higher values of ∑DFPMAA_250_ can compensate the requirements of “glycogen degradation” in the microbe community. In other words, with diversified availability of carbons *via* enhanced metabolic potentials as implied by high ∑DFPMAA_250_ scores, bacteria can thrive with less dependence upon glycogen degradation. It would be interesting to determine if simultaneous additions of glycan-rich foods with *CBM588* could elicit any synergistic effects.

In this study, we used *CBM588* instead of the more common *Lactobacilli*-based probiotics because its safety is well established^35^. Reports of its efficacy against enterohemorrhagic *Escherichia coli* O157:H7^36^ and *Clostridium difficile*^37^ also increased our confidence in its use in immunocompromised patients. In a rat model following SBT, Price *et al*. found that rejection and graft-versus-host disease are associated with shifts in gut microbiota toward potentially pathogenic organisms^38^, which can be ameliorated using probiotics^39^. In humans, Oh *et al*. indicated that the presence of the *Enterobacteriaceae* family significantly increases during episodes of rejection after SBT^14^. Bacterial compositions could also be affected by the presence of ileostomy and the availability of oxygen after a transplantation^40^. In our study, the most notable alterations were decreases in *Enterobacteriaceae* and increases in *Bacteroidaceae* after the 1-month ingestion of *CBM588* in patients (Fig. 1E). With the known association between the *Enterobacteriaceae* family and graft rejection^14^, our results would support the use of *CBM588* to improve the survival of small intestine allografts. The decrease in *Enterobacteriaceae per se* would in addition imply a lower likelihood of infection by potential pathogens in the family. Risks of rejection and infection might accordingly be minimized from the use of *CBM588*. Although number of patients in our cohort was limited, our data did reveal the time-dependent divergence of microbiota and concordant change of carbohydrate metabolism after *CBM588* ingestion. Further large-scale investigations are warranted. The developed scoring system detailed herein could readily provide objective and quantitative readouts to facilitate the establishment of clinical reasoning behind adopting probiotics in the care of SBT recipients.

## Methods

### Sample collection and ethics approval

All patients received SBT 6 months before enrolment into the study (Table 1). Attendees took *CBM588* (1.5 × 10^9^ CFU/day) daily for 1 month (Fig. 1A). Stool and/or blood samples were collected before, 1 week, and 1 month after *CBM588* ingestion. The experimental protocol was approved by the Institutional Review Board of Far Eastern Memorial Hospital, New Taipei City. The study was conducted in accordance with the relevant guidelines. Informed consent was obtained from patients directly or from their parents if attendees were younger than 18 years old.

### Software and hardware

All analyses were performed on a 2013 Mac Pro equipped with 3.7-GHz Quad-Core Intel Xeon E5, 64 GB of memory, and 2 AMD FirePro D700 6 GB graphics cards. Inputs and outputs of various specialized packages were glued with Python 2.7 scripts.

### Next-generation sequencing

All sequencing libraries including 16S rRNA, shotgun metagenomes, and *CBM588* genome were constructed and sequenced using commercial tools. The 16S libraries were sequenced on Illumina MiSeq as 2 × 300 bp paired-end readings, whereas the shotgun fecal metagenomes and *CBM588* genome were determined on Illumina NextSeq as 2 × 150 bp paired-end readings.

### Operational taxonomic units

Raw reads (Supplementary Fig. S1A) carrying primer sequences were trimmed using a self-developed script. Results were paired using PEAR^41^ and filtered using USEARCH^15^. Pooled sequences were used to define operational taxonomic units (OTUs) by USEARCH with default criteria. Saturation curves of distinct OTUs (Supplementary Fig. S1B) were plotted as means of 10 random selections with increasing numbers of paired reads. Ten rarefied subsets of reads in the format of mapped OTUs were prepared by giving each the same number of reads before downstream analyses were conducted.

### Diversities in Hill numbers

Hill numbers define a diversity profile with a parameter *q* formulated as follows, where *S* is “species equivalent,” *f* represents frequency of “species equivalent,” and *q* denotes the parameter.

$${D}_{q}={({\sum }_{s}{f}^{q})}^{1/(1-q)}$$

With increasing values of *q*, more weights are given to the more abundant species equivalent. In this study, OTUs (Fig. 1B, Supplementary Figs S3A, and S4), or dbCAN-defined hidden Markov models (HMMs)^23^ (Figs 3D and 4D) were used as species equivalents. At *q* = 0, species equivalents were counted without considering their normalized frequencies. We employed this zero-parameterized Hill number as the definition for α diversity^42^. At *q* = 1, counts were proportional to their normalized frequencies (i.e., Shannon diversity), whereas at *q* = 2, only dominant species equivalents were counted. A deeper slope of the curve represents an unevener distribution of relative abundances of species equivalents.

### Principal component analysis

Decomposition with principal component analysis (PCA) was performed with scikit-learn in Python^43^. Values were Hellinger-transformed before analyses^17^. OTUs and KEGG-defined pathways^22^ were used as variables to decompose 16S rRNA and metagenome functions, respectively.

### Selection of signature phyla and families

OTUs were taxonomically classified using USEARCH^15^ and a SILVA-based reference^18^. 16S rRNA reads were given the same taxonomic designations as the associated OTUs. Mixed linear models^19^ (MLMs) were adopted to identify signature phyla which best discriminated *CBM588* effects among at least half of the rarefied data sets. Support vector classification^20^ (SVC) and logistic regression^21^ (LR) were used to select signature families linked to *CBM588* exposure. Only those families that were in agreement in both SVC and LR models among at least half of the rarefied data sets were taken. Parameters were optimized by leave-one-out cross-validations.

### 16S rRNA-inferred metagenome functions

Piphillin^10^ was used to extrapolate metagenomic functions to KEGG pathways^22^ with 16S rRNA sequences. In addition to PCA analyses, KEGG pathways were also subjected to signature selections with SVC^20^ and LR^21^ models. Only those picked by both models among at least half of the rarefied data sets would be accepted. Model parameters were optimized with leave-one-out cross-validations.

### Domain fractions for reads of shotgun metagenomes and bacteria genomes

Raw reads (Supplementary Fig. S1C) were paired using PEAR^41^ with quality control defaults. Bowtie 2^44^ was used to filter out human sequences for reads of human origin. Genes were predicted using FragGeneScan^45^. CAZy-associated^28^ HMMs were downloaded from dbCAN^23^ for HMMER scanning^46^ upon PEAR-assembled reads, where unassembled reads were excluded. “hmmsearch” with Z-value adjustment to 585 was used instead of “hmmscan” to increase scanning efficiency. Calculations of domain fractions are specified below.

### Domain fractions per million amino acids 250

Domain fractions were determined by hmmscan-parser.sh as downloaded from dbCAN^23^, but minimal changes to switch parameters were made to reflect the use of “hmmsearch” instead of “hmmscan.” Other criteria, including *p* values and a minimal domain fraction of 0.3 as defined by dbCAN, were not altered. For a given domain, summed domain fractions normalized by counts of amino acid inputs and 250 base pairs were designated as “domain fractions per million amino acids per 250 nucleotides” or DFPMAA_250_ (Supplementary Fig. S2B). Python scripts for the demonstration purpose is available in the Supplementary Information.

### HUMAnN2 validation

Evaluations were repeated 5 times against the MetaCyc^25^ database. Each run was conducted upon a rarefied data set of 5 million randomly picked paired reads of each sample from the original shotgun data sets (Supplementary Fig. S1C). There are 11 instance pathways in the MetaCyc^25^ “Glycan Degradation” Class, including (1,4)-β-D-xylan degradation, cellulose degradation I, chondroitin sulfate degradation, dermatan sulfate degradation, glycogen degradation I, glycogen degradation II, homogalacturonan degradation, L-arabinan degradation, pectin degradation I, starch degradation I, and xyloglucan degradation I. Abundance of “glycogen degradation II” was discarded because the pathway is restricted to eukaryotes.

### *CBM588* genome sequencing

The construction of libraries and next-generation sequencing were contracted to commercial service providers. Raw reads (Supplementary Fig. S1D) were assembled by SPAdes^47^ into contigs and scaffolds to confirm the probiotic identity by *in silico* polymerase chain reaction^48^ to find corresponding 16S sequences with 5′-TCGTCGGCAGCGTCAGATGTGTATAAGAGACAGCCTACGGGNGGCWGCAG-3′ and 5′-GTCTCGTGGGCTCGGAGATGTGTATAAGAGACAGGACTACHVGGGTATCTAATCC-3′ primers (Supplementary Fig. S1E). For calculations of DFPMAA_250_ raw reads were processed as fecal shotgun metagenomes without contig or scaffold assembling.

### DFPMAA_250_ for bacteria genomes

Shotgun genomes of bacteria were found from the data sets of Köser *et al*.^27^, whereas the *CBM588* genome was prepared as described in the previous section. Shorter versions of *CBM588* shotgun sequences (Supplementary Fig. S7) were simulated by using random trimmings of 5′ and 3′ ends of paired full-length reads. Only those bacteria with mean lengths of paired reads of over 187.6 bp were subjected to DFPMAA_250_ analyses. Gene predictions and protein domain scanning were conducted in the same manner for fecal shotgun metagenomes but without the use of Bowtie 2 filters (Supplementary Fig. S2B).

## Supplementary information

Supplementary Information

## Data Availability

All raw sequencing data generated in this study have been submitted to the European Nucleotide Archive (https://www.ebi.ac.uk/ena) under accession number PRJEB27661.

## References

[1] Alexandre, A. *et al*. JRC F7 - Knowledge for Health and Consumer Safety, The Human Gut Microbiota: Overview and analysis of the current scientific knowledge and possible impact on healthcare and well-being. *Publications Office of the European Union, Luxembourg*, EUR - Scientific and Technical Research Reports, 10.2760/17381 (2018).

[2] Stephen AM, Cummings JH (1980). The microbial contribution to human faecal mass. J Med Microbiol.

[3] Chung H (2012). Gut immune maturation depends on colonization with a host-specific microbiota. Cell.

[4] Chua HH (2018). Intestinal Dysbiosis Featuring Abundance of Ruminococcus gnavus Associates With Allergic Diseases in Infants. Gastroenterology.

[5] Wong JM, de Souza R, Kendall CW, Emam A, Jenkins DJ (2006). Colonic health: fermentation and short chain fatty acids. J Clin Gastroenterol.

[6] Li X, Shimizu Y, Kimura I (2017). Gut microbial metabolite short-chain fatty acids and obesity. Biosci Microbiota Food Health.

[7] Weinstock GM (2012). Genomic approaches to studying the human microbiota. Nature.

[8] Olsen GJ, Lane DJ, Giovannoni SJ, Pace NR, Stahl DA (1986). Microbial ecology and evolution: a ribosomal RNA approach. Annu Rev Microbiol.

[9] Langille MG (2013). Predictive functional profiling of microbial communities using 16S rRNA marker gene sequences. Nat Biotechnol.

[10] Iwai S (2016). Piphillin: Improved Prediction of Metagenomic Content by Direct Inference from Human Microbiomes. PLoS One.

[11] Quince C, Walker AW, Simpson JT, Loman NJ, Segata N (2017). Shotgun metagenomics, from sampling to analysis. Nat Biotechnol.

[12] Sudan D (2014). The current state of intestine transplantation: indications, techniques, outcomes and challenges. Am J Transplant.

[13] Taur Y (2014). The effects of intestinal tract bacterial diversity on mortality following allogeneic hematopoietic stem cell transplantation. Blood.

[14] Oh PL (2012). Characterization of the ileal microbiota in rejecting and nonrejecting recipients of small bowel transplants. Am J Transplant.

[15] Edgar RC (2010). Search and clustering orders of magnitude faster than BLAST. Bioinformatics.

[16] Hill MO (1973). Diversity and evenness: a unifying notation and its consequences. Ecology.

[17] Legendre P, Gallagher ED (2001). Ecologically meaningful transformations for ordination of species data. Oecologia.

[18] Quast C (2013). The SILVA ribosomal RNA gene database project: improved data processing and web-based tools. Nucleic Acids Res.

[19] Lindstrom MJ, Bates DM (1988). Newton—Raphson and EM algorithms for linear mixed-effects models for repeated-measures data. Journal of the American Statistical Association.

[20] Chang C-C, Lin C-J (2011). LIBSVM: A library for support vector machines. ACM transactions on intelligent systems and technology (TIST).

[21] Fan R-E, Chang K-W, Hsieh C-J, Wang X-R, Lin C-J (2008). LIBLINEAR: A library for large linear classification. Journal of machine learning research.

[22] Kanehisa M, Goto S (2000). KEGG: kyoto encyclopedia of genes and genomes. Nucleic Acids Res.

[23] Yin Y (2012). dbCAN: a web resource for automated carbohydrate-active enzyme annotation. Nucleic Acids Res.

[24] Franzosa EA (2018). Species-level functional profiling of metagenomes and metatranscriptomes. Nature methods.

[25] Caspi R (2013). The MetaCyc database of metabolic pathways and enzymes and the BioCyc collection of Pathway/Genome Databases. Nucleic acids research.

[26] Wilson WA (2010). Regulation of glycogen metabolism in yeast and bacteria. FEMS microbiology reviews.

[27] Koser CU (2014). Rapid single-colony whole-genome sequencing of bacterial pathogens. J Antimicrob Chemother.

[28] Lombard V, Golaconda Ramulu H, Drula E, Coutinho PM, Henrissat B (2014). The carbohydrate-active enzymes database (CAZy) in 2013. Nucleic Acids Res.

[29] Larsen PE (2011). Predicted Relative Metabolomic Turnover (PRMT): determining metabolic turnover from a coastal marine metagenomic dataset. Microb Inform Exp.

[30] Heinken A, Thiele I (2015). Systems biology of host–microbe metabolomics. Wiley Interdisciplinary Reviews: Systems Biology and Medicine.

[31] Xu D, Nussinov R (1998). Favorable domain size in proteins. Folding and Design.

[32] Eilam O (2014). Glycan degradation (GlyDeR) analysis predicts mammalian gut microbiota abundance and host diet-specific adaptations. MBio.

[33] Marcobal A (2011). Bacteroides in the infant gut consume milk oligosaccharides via mucus-utilization pathways. Cell Host Microbe.

[34] Sonnenburg ED (2010). Specificity of polysaccharide use in intestinal bacteroides species determines diet-induced microbiota alterations. Cell.

[35] Isa K (2016). Safety assessment of the Clostridium butyricum MIYAIRI 588(R) probiotic strain including evaluation of antimicrobial sensitivity and presence of Clostridium toxin genes *in vitro* and teratogenicity *in vivo*. Hum Exp Toxicol.

[36] Takahashi M (2004). The effect of probiotic treatment with Clostridium butyricum on enterohemorrhagic Escherichia coli O157:H7 infection in mice. FEMS Immunol Med Microbiol.

[37] Woo TD (2011). Inhibition of the cytotoxic effect of Clostridium difficile *in vitro* by Clostridium butyricum MIYAIRI 588 strain. J Med Microbiol.

[38] Price BA (1993). The effect of rejection and graft-versus-host disease on small intestinal microflora and bacterial translocation after rat small bowel transplantation. Transplantation.

[39] Zhou HJ, Yin L, Chen CQ, Shi MM, Zhang MJ (2010). Administration of probiotics reduces bacterial translocation after intestinal transplantation in rats. Transplant Proc.

[40] Hartman AL (2009). Human gut microbiome adopts an alternative state following small bowel transplantation. Proc Natl Acad Sci USA.

[41] Zhang J, Kobert K, Flouri T, Stamatakis A (2014). PEAR: a fast and accurate Illumina Paired-End reAd mergeR. Bioinformatics.

[42] McCoy CO, Matsen FAt (2013). Abundance-weighted phylogenetic diversity measures distinguish microbial community states and are robust to sampling depth. PeerJ.

[43] Pedregosa F (2011). Scikit-learn: Machine Learning in Python. J. Mach. Learn. Res..

[44] Langmead B, Salzberg SL (2012). Fast gapped-read alignment with Bowtie 2. Nat Methods.

[45] Rho M, Tang H, Ye Y (2010). FragGeneScan: predicting genes in short and error-prone reads. Nucleic Acids Res.

[46] Eddy SR (2011). Accelerated Profile HMM Searches. PLoS Comput Biol.

[47] Bankevich A (2012). SPAdes: a new genome assembly algorithm and its applications to single-cell sequencing. J Comput Biol.

[48] Kalendar R, Khassenov B, Ramankulov Y, Samuilova O, Ivanov KI (2017). FastPCR: An in silico tool for fast primer and probe design and advanced sequence analysis. Genomics.

