# Supplementary material for: Integrated Counts of Carbohydrate-Active Protein Domains as Metabolic Readouts to Distinguish Probiotic Biology and Human Fecal Metagenomes
Source: Sci Rep. 2019 Nov 14;9:16836. doi: 10.1038/s41598-019-53173-7 (PMC6856387; doi:10.1038/s41598-019-53173-7)
Supplement: Supplementary file 1 — Supplementary Information [file 41598_2019_53173_MOESM1_ESM.pdf]

Integrated Counts of Carbohydrate-Active Protein Domains as Metabolic Readouts to Distinguish Probiotic Biology and Human Fecal Metagenomes

Hong-Hsing Liu, Yu-Chen Lin, Chen-Shuan Chung, Kevin Liu, Ya-Hui Chang, Chung-Hsiang Yang, Yun Chen, Yen-Hsuan Ni, and Pi-Feng Chang

Supplementary Figure S1. Summaries of next-generation sequencing results. (A) Read numbers of 16S rRNA amplicons from patients at all three time points were tabulated. **(B)** Saturating curves of distinct OTUs as averaged from 10 random selections at given depths were plotted for all patients. **(C)** Read numbers of shotgun metagenome sequencing from samples before and 1-month after *CBM588* ingestions were summarized. **(D)** Read numbers of the *CBM588* shotgun genome were listed. **(E)** 16S sequences as extracted from *CBM588* shotgun genome sequences were shown.

Supplementary Figure S2. Pipelines in the study. (A) 16S rRNA amplicons were categorized into OTUs and 10 rarefied datasets were prepared for diversity analyses, taxonomic signature selections, and metagenome function inferences. **(B)** Domain fractions per million amino acids per 250 nucleotides ($DFPMAA_{250}$) from shotgun sequences was defined.

Supplementary Figure S3. Taxonomic evaluations of fecal microbiota associated with *CBM588* ingestions for 1 week. (A) OTU diversities at 1 week were evaluated with Hill numbers. Both dashed (before) and solid (1-week after) curves nearly overlapped, implying absences of dominant OTUs after *CBM588* ingestions. At q = 0, Hill numbers equal α diversity, which increased from 226.3 ± 2.6 to 251.0 ± 3.2 (SD) as estimated from 10 rarefied datasets. **(B)** 1-week exposure of *CBM588* brought about relatively minor shifts of OTU profiles on principal component analyses for most patients. Samples from the same patient were designated with the same color. Circles (before) and triangles (1-week after) were close-by for most patients. **(C)** Percentages of five phyla, including *Actinobacteria, Bacteroidetes, Firmicutes, Proteobacteria, and Verrucomicrobia* showed discernible shifts at 1-week after *CBM588* ingestions, which were compatible with 1-month evaluations. **(D)** Four families, including *Bacteroidaceae*, *Enterobacteriaceae*, *Veillonellaceae,* and *Clostridiaceae* had noticeable changes after 1-week exposures to *CBM588*, which were concordant with 1-month assessments.

Supplementary Figure S4. OTU diversities in Hill numbers. (A) Samples before or 1-week after *CBM588* ingestions were plotted for all 10 rarefied datasets. Both curves almost overlapped. **(B)** Samples before or 1-month after *CBM588* ingestions were plotted for all 10

rarefied datasets. Few distinctions were found for the two curves.

Supplementary Figure S5. Principal component analyses of OTUs. (A) Samples before or 1-week after *CBM588* ingestions were plotted for all 10 rarefied datasets. Shifts were relatively minor between the pre-exposure samples (circles) and the post-exposure samples (triangles). Different patients were designated with different colors. **(B)** Samples before or 1-month after *CBM588* ingestions were plotted for all 10 rarefied datasets. Shifts were apparent after *CBM588* administrations (circles *vs.* triangles of the same color).

Supplementary Figure S6. Principal component analyses of 16S rRNA-inferred KEGG functional pathways. Most patients showed distinct shifts between samples before (circles) and 1-month after (triangles) *CBM588* ingestions.

Supplementary Figure S7. $\sum DFPMAA_{250}$ *vs.* mean read lengths of shotgun bacteria genomes. (A) Shotgun genome sequences of *CBM588* were randomly and systematically shortened in sets of 10 rarefactions to evaluate impacts of mean read lengths to the calculations of $\sum DFPMAA_{250}$ values. DFPMAA without the 250 subscript denotes values without normalization to 250 nucleotides. It was found the mean read lengths could be shortened up to 187.6 bp without apparent deviations of $\sum DFPMAA_{250}$ estimates. **(B)** The mean read lengths of 10 rarefactions from all bacteria evaluated were tabulated. For fair comparisons the mean read lengths of the *CBM588* genome were artificially made the shortest.

Supplementary Python scripts. Scripts for UNIX-like platforms are provided to demonstrate the calculation of $\sum DFPMAA_{250}$. Please find README.txt to setup a required environment. Two datasets and relevant Hidden Markov Models will be downloaded from the cloud automatically.

Supplementary Fig. S1

A

2 x 300 bp	P1			P2			P3			P4		
	Before	1 Week	1 Month	Before	1 Week	1 Month	Before	1 Week	1 Month	Before	1 Week	1 Month
16S rDNA	72,227	79,374	82,155	86,270	80,983	429,475	494,020	446,119	446,297	356,653	383,552	446,393

2 x 300 bp	P5			P6			P7		
	Before	1 Week	1 Month	Before	1 Week	1 Month	Before	1 Week	1 Month
16S rDNA	511,849	313,626	348,420	401,686	484,825	459,553	391,518	313,261	527,496

B

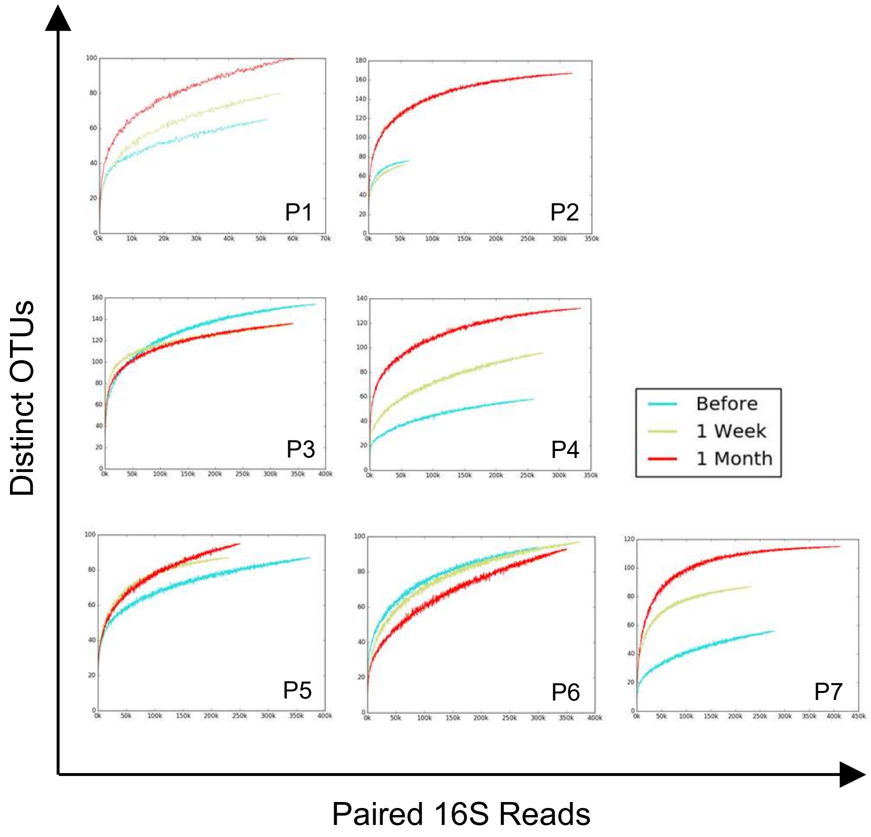

C

2 x 150 bp	P2	
	Before	1 Month
Shotgun	51,070,300	55,507,257

2 x 150 bp	P3	
	Before	1 Month
Shotgun	22,223,651	38,042,942

2 x 150 bp	P5	
	Before	1 Month
Shotgun	38,842,344	50,317,113

D

2 x 150 bp	*CBM588*
Shotgun	425,793

E

> *CBM588* 16S

TGTTTGCTCCCCACGCTTTCGAGCCTCAGTGTCAGTTACAGTCCAGAAAGGCGCCTTCGCCACTGGTAT
TCTTCCTAATCTCTACGCATTTCACCGCTACACTAGGAATTCTCCTTTCCTCTCCTGCACTCTAGATAT
CCAGTTTGGAATGCAGCACCCAGGTTAAGCCCGGGTATTTCACATCCCACTTAAATATCCACCTACGCT
CCCTTTACGCCCAGTAAATCCGGACAACGCTTGCCACCTACGTATTACCGCGGCTGCTGGCACGTAGTT
AGCCGTGGCTTCCTCCTTAGGTACCGTCATTATCGTCCCTAAAGACAGAGCTTTACAATCCGAAGACCG
TCATCACTCACGCGGCGTTGCTGCATCAGGGTTTCCCCCATTGTGCAATATTCCCCA

Supplementary Fig. S2

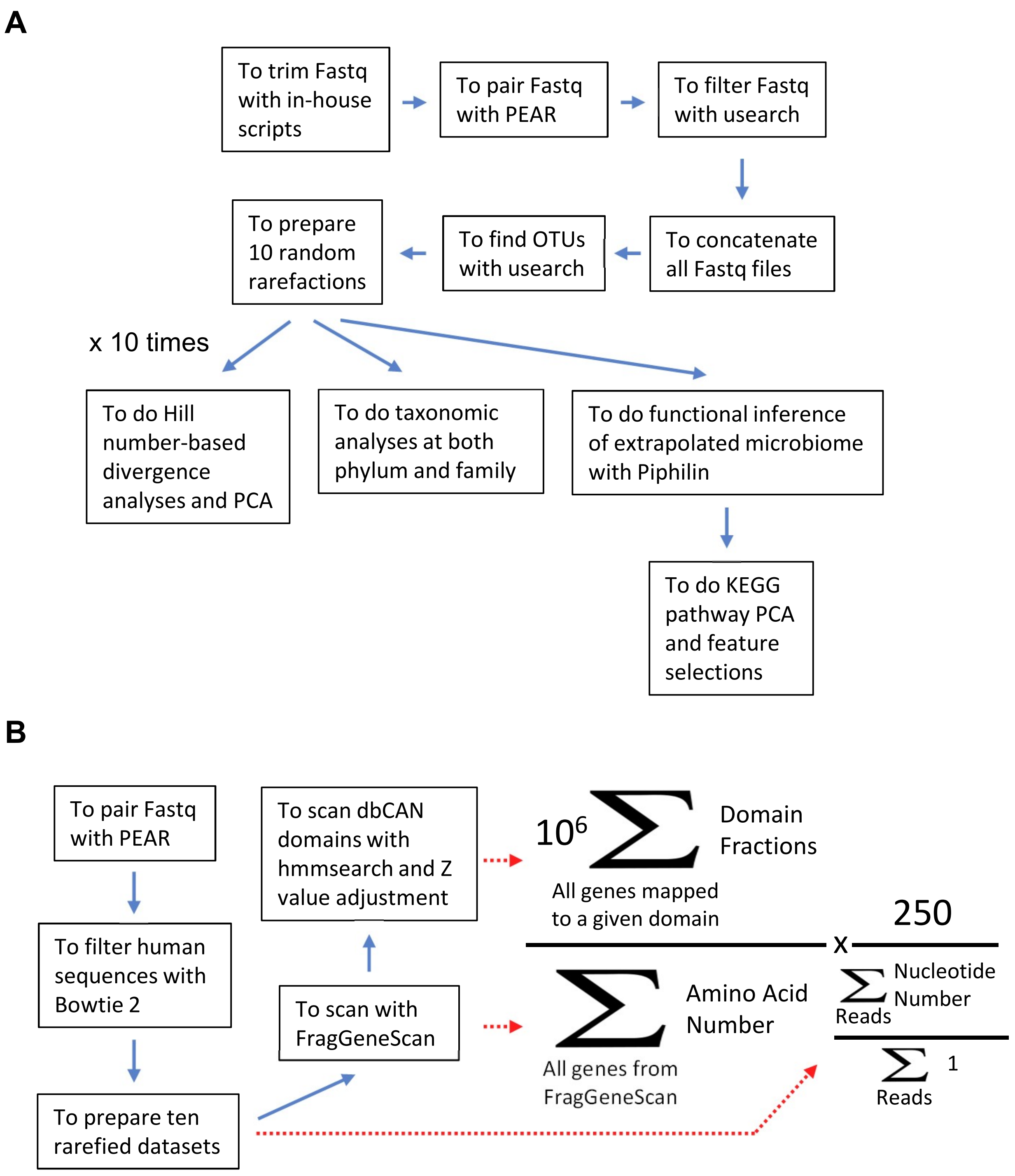

Domain Fractions per Million Amino Acids per 250 Nucleotides ($DFPMAA_{250}$)

Supplementary Fig. S3

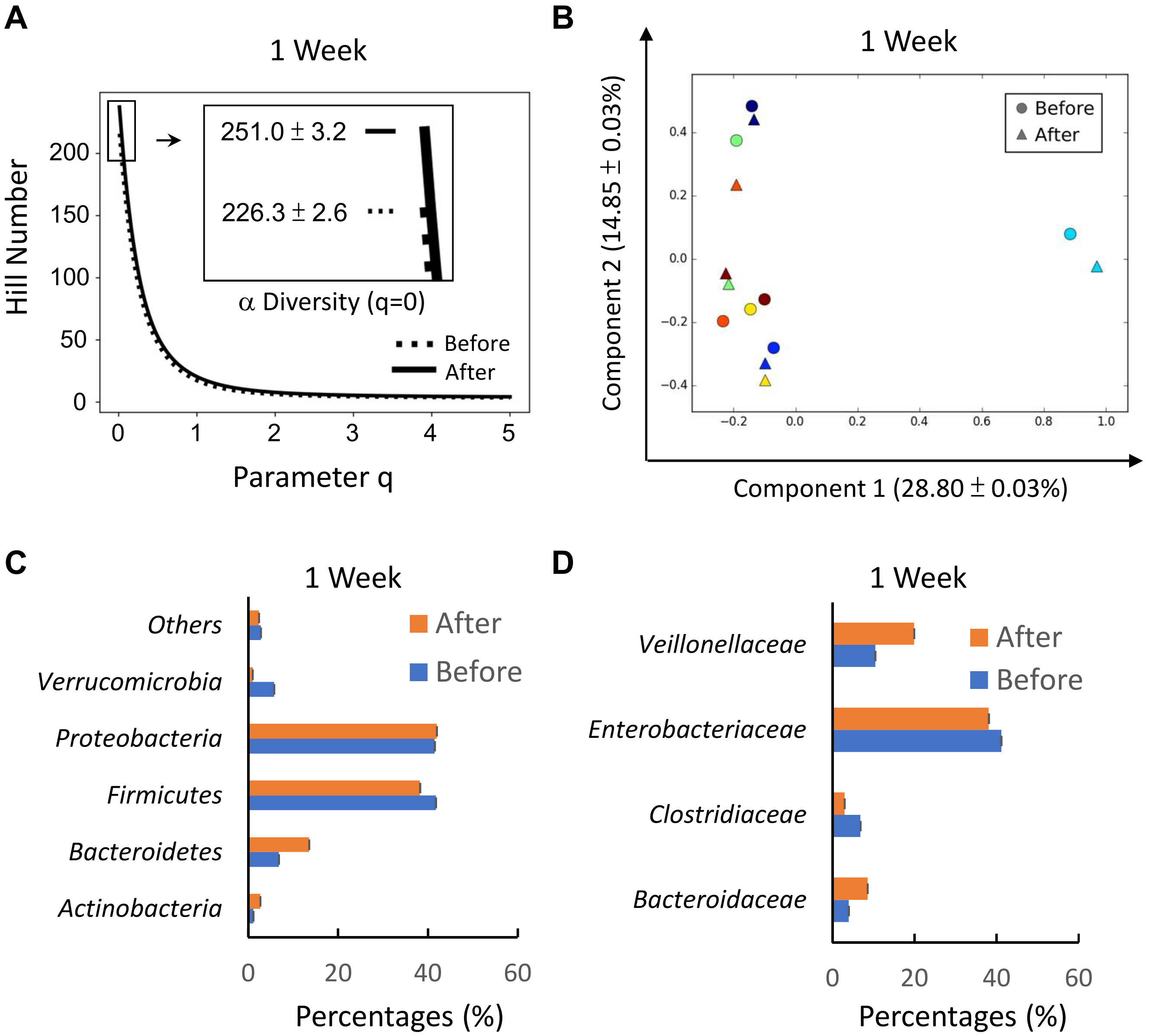

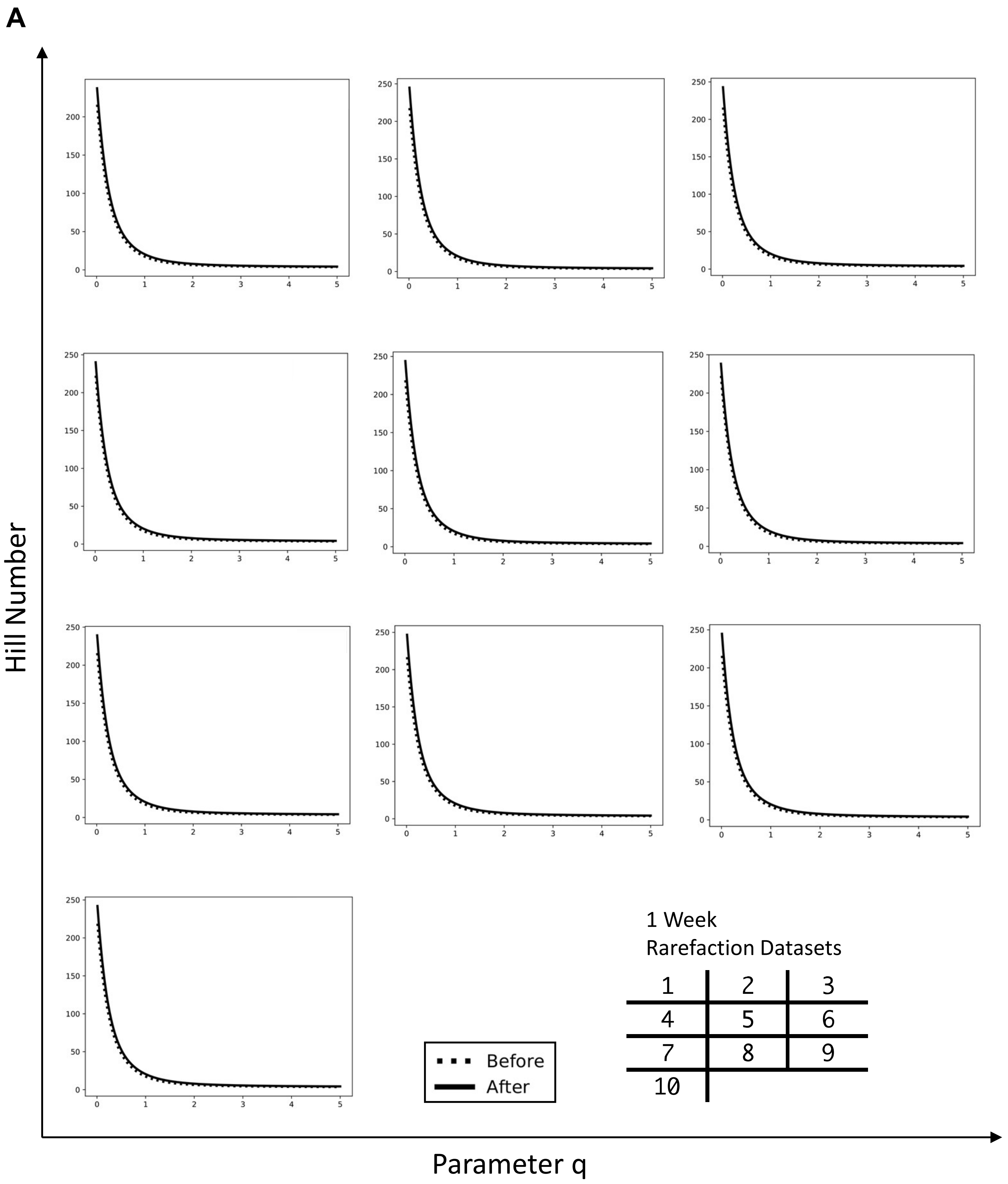
A
Hill Number
Parameter q
Before
After
1 Week
Rarefaction Datasets
1
2
3
4
5
6
7
8
9
10

B

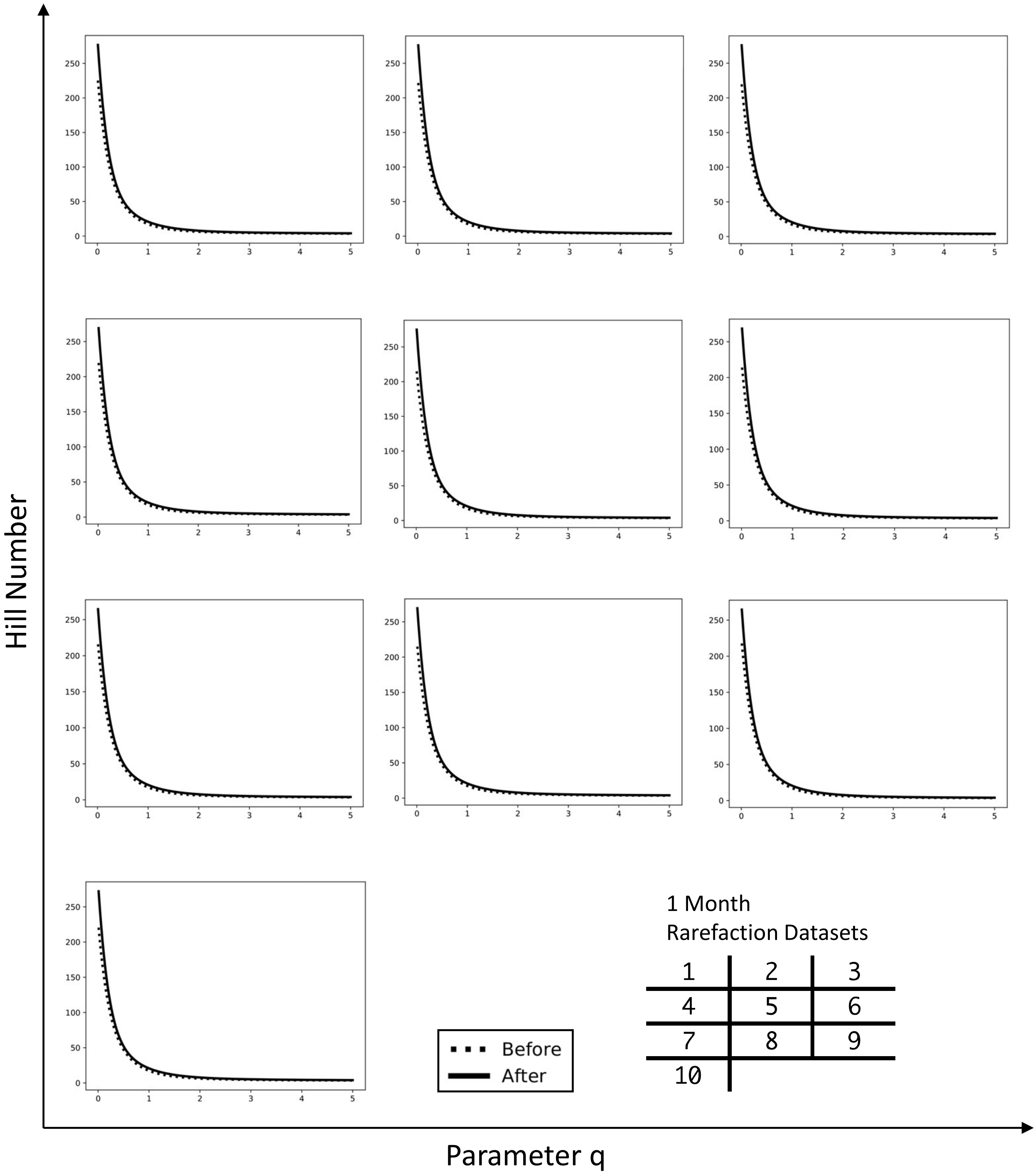
Hill Number
Parameter q
Before
After
1 Month
Rarefaction Datasets
1 2 3
4 5 6
7 8 9
10
0 50 100 150 200 250
0 1 2 3 4 5

Supplementary Fig. S5

A

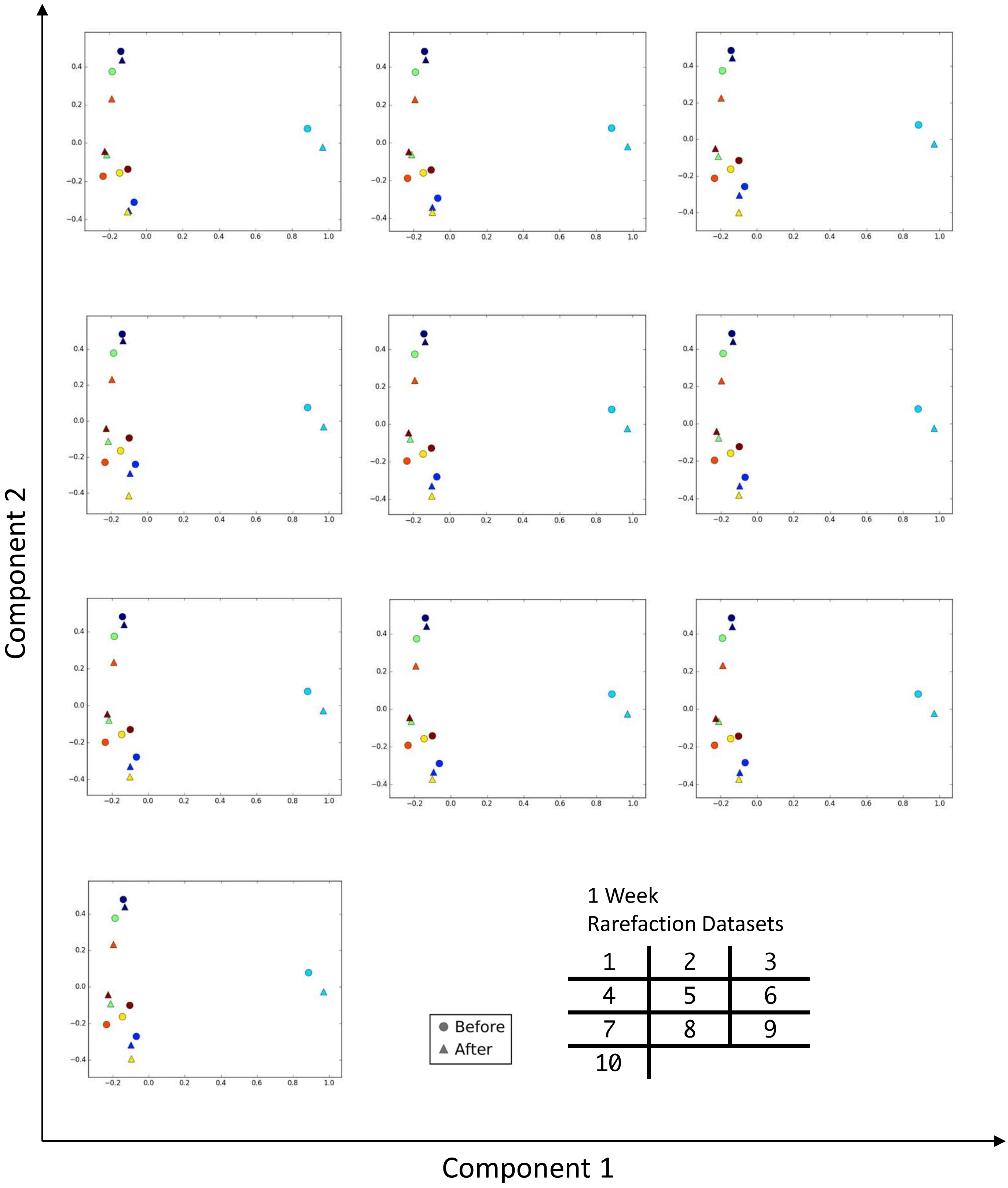

B

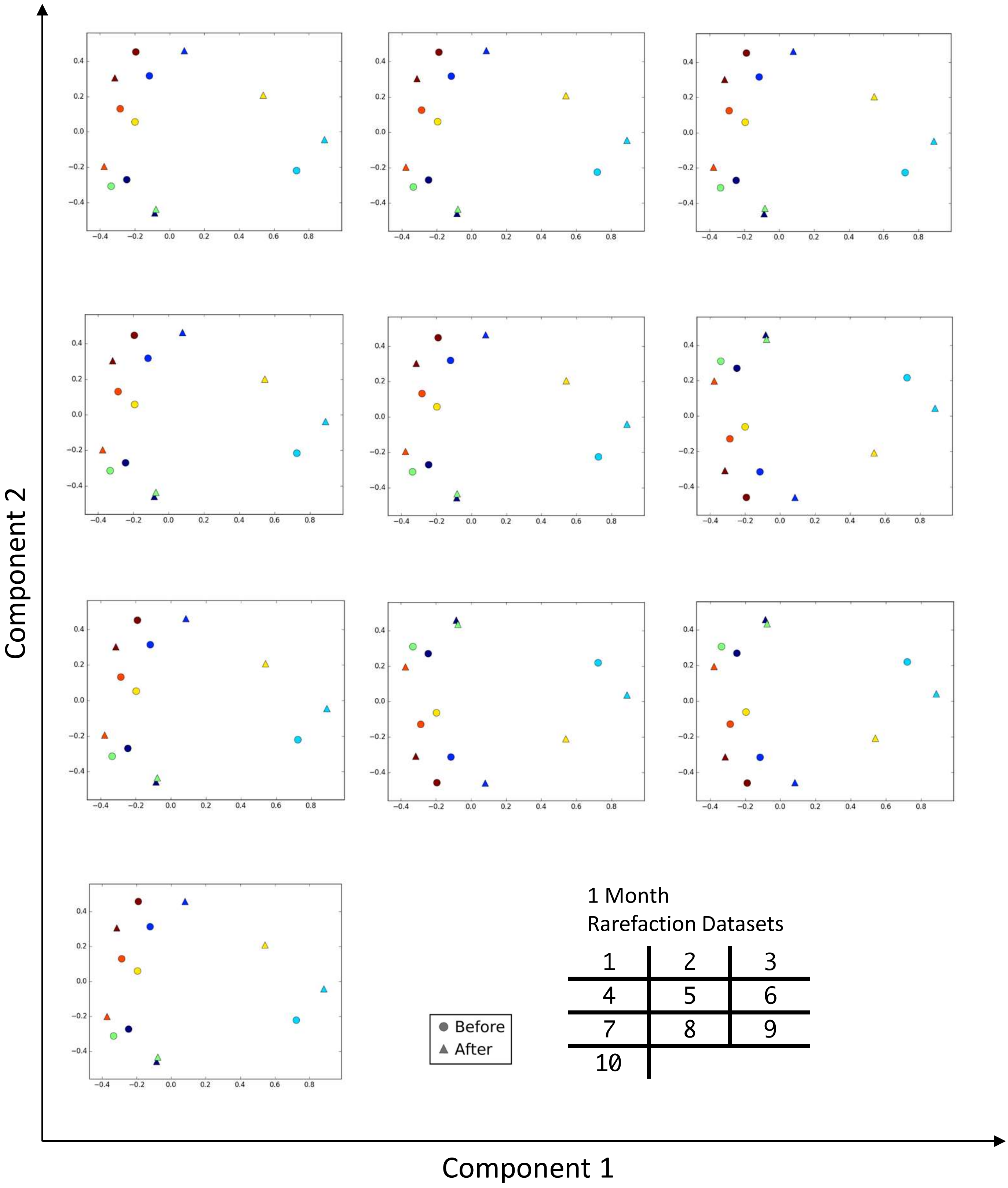

Component 2
Component 1
Before
After
1 Month
Rarefaction Datasets
1 2 3
4 5 6
7 8 9
10

Supplementary Fig. S6

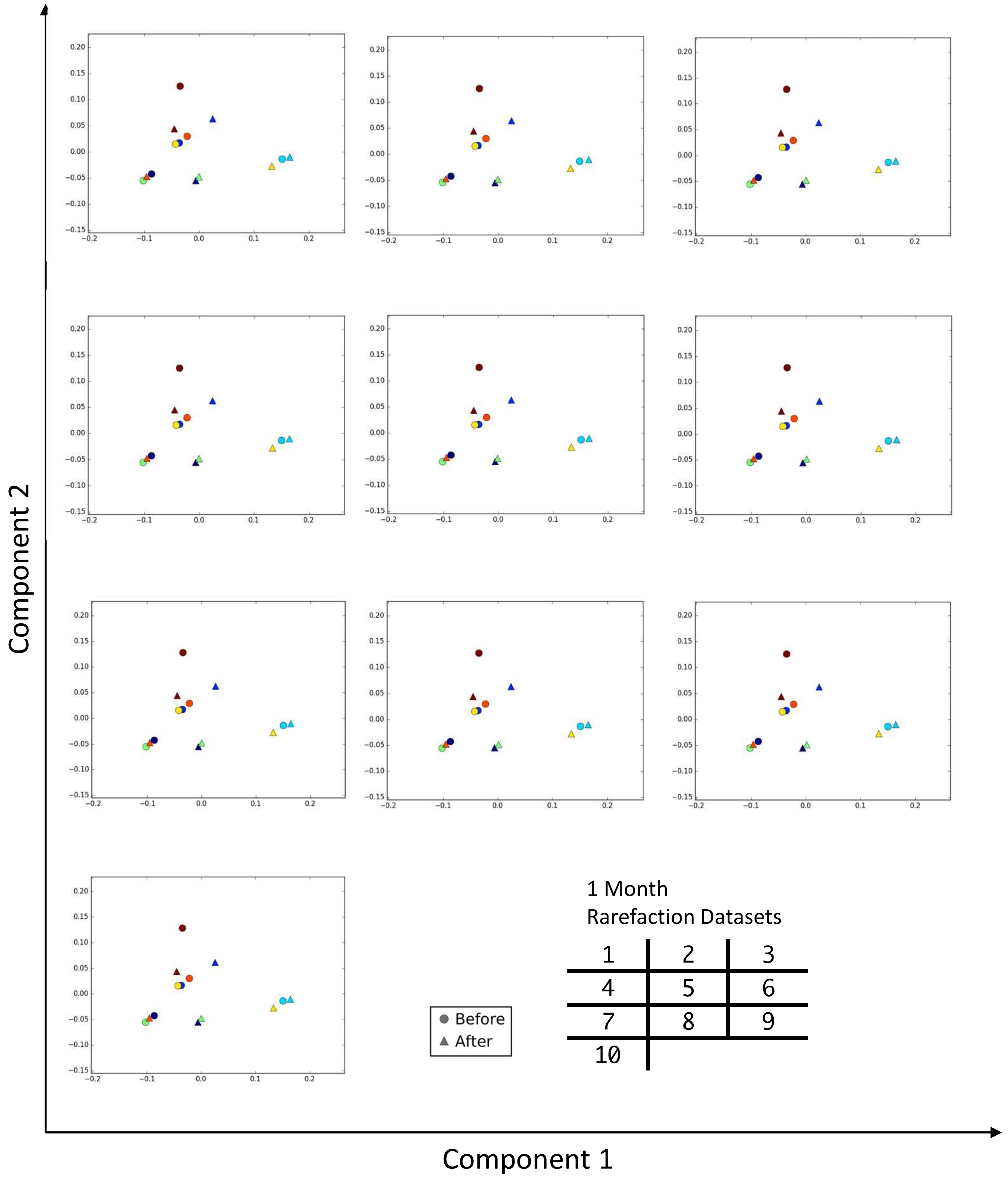

A

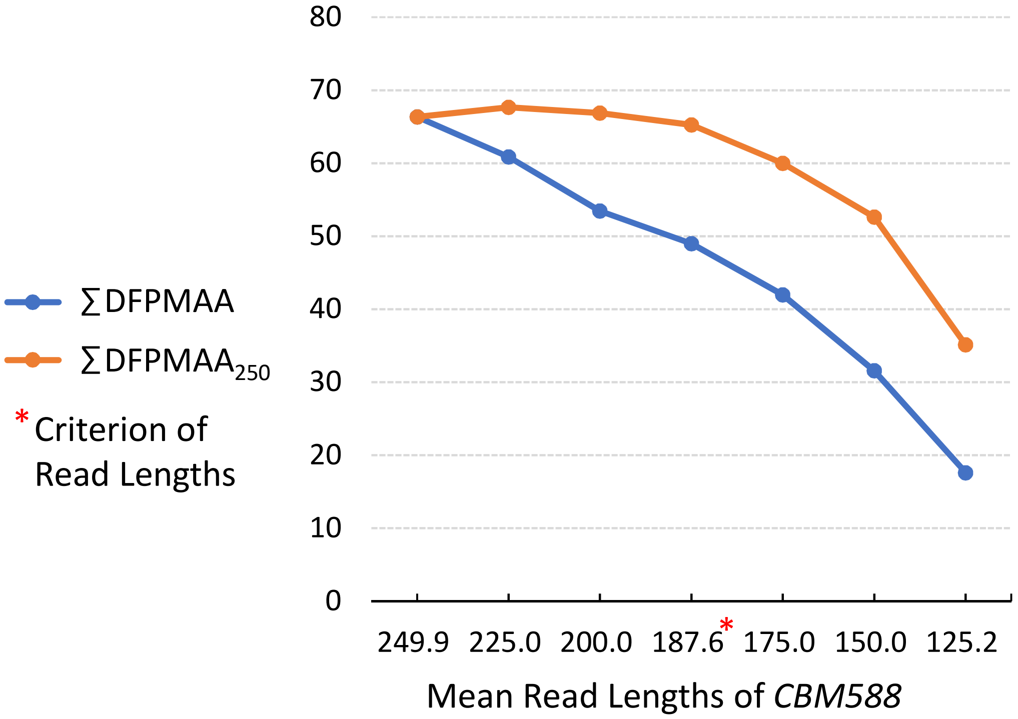

B

Mean Read Lengths of Rarefactions

*CBM588**	*ATCC 17978*	*ATCC 700802*	*ATCC 700926*	*ATCC 51907*	*ATCC 700721*	*NCTC 11192*	*Cam. Salmonella*	*ATCC BAA-611*	*ATCC BAA-334*
187.6	195.1	211.3	189.8	188.2	196.1	196.1	191.8	191.5	189.4
187.6	195.1	210.8	189.7	188.1	196.1	195.9	191.8	191.5	189.0
187.6	194.9	210.6	189.3	188.3	196.1	196.3	191.4	191.5	189.0
187.6	194.9	210.9	189.6	188.6	195.9	196.1	192.0	191.7	189.1
187.6	194.8	211.3	189.3	188.2	196.1	196.0	191.8	191.4	189.0
187.6	194.7	211.0	189.9	188.0	195.9	195.8	191.7	191.5	189.1
187.6	194.9	211.3	189.6	188.4	195.7	195.9	191.8	191.8	189.5
187.6	194.8	211.0	190.0	188.5	195.7	196.0	191.8	191.5	189.5
187.6	194.6	210.9	189.8	188.8	195.6	196.1	192.2	191.2	189.5
187.6	194.9	211.1	189.9	188.5	195.6	196.1	192.1	191.4	189.0

*Before calculations of $DFPMAA_{250}$ read lengths of *CBM588* were randomly shorted by a factor 0.75 from the original set.

**There were 90,164 reads per strain in each rarefaction.
